# A Novel Optimization Technique to Improve Gas Recognition by Electronic Noses Based on the Enhanced Krill Herd Algorithm

**DOI:** 10.3390/s16081275

**Published:** 2016-08-12

**Authors:** Li Wang, Pengfei Jia, Tailai Huang, Shukai Duan, Jia Yan, Lidan Wang

**Affiliations:** College of Electronic and Information Engineering, Southwest University, Chongqing 400715, China; m18202373438@163.com (L.W.); 18580465830@163.com (T.H.); duansk@swu.edu.cn (S.D.); yanjia119@163.com (J.Y.); ldwang@swu.edu.cn (L.W.)

**Keywords:** EKH, electronic nose, optimization algorithm, decision weighting factor, indoor pollutant gas

## Abstract

An electronic nose (E-nose) is an intelligent system that we will use in this paper to distinguish three indoor pollutant gases (benzene (C_6_H_6_), toluene (C_7_H_8_), formaldehyde (CH_2_O)) and carbon monoxide (CO). The algorithm is a key part of an E-nose system mainly composed of data processing and pattern recognition. In this paper, we employ support vector machine (SVM) to distinguish indoor pollutant gases and two of its parameters need to be optimized, so in order to improve the performance of SVM, in other words, to get a higher gas recognition rate, an effective enhanced krill herd algorithm (EKH) based on a novel decision weighting factor computing method is proposed to optimize the two SVM parameters. Krill herd (KH) is an effective method in practice, however, on occasion, it cannot avoid the influence of some local best solutions so it cannot always find the global optimization value. In addition its search ability relies fully on randomness, so it cannot always converge rapidly. To address these issues we propose an enhanced KH (EKH) to improve the global searching and convergence speed performance of KH. To obtain a more accurate model of the krill behavior, an updated crossover operator is added to the approach. We can guarantee the krill group are diversiform at the early stage of iterations, and have a good performance in local searching ability at the later stage of iterations. The recognition results of EKH are compared with those of other optimization algorithms (including KH, chaotic KH (CKH), quantum-behaved particle swarm optimization (QPSO), particle swarm optimization (PSO) and genetic algorithm (GA)), and we can find that EKH is better than the other considered methods. The research results verify that EKH not only significantly improves the performance of our E-nose system, but also provides a good beginning and theoretical basis for further study about other improved krill algorithms’ applications in all E-nose application areas.

## 1. Introduction

An electronic nose (E-nose) is a device composed of a gas sensor array and an artificial intelligence algorithm. It is effective in dealing with odor analysis problems [1,2,3], and during the past decades, much work has been done to prove the efficiency of E-nose technology in many fields such as environmental monitoring [4,5], food engineering [6,7,8], disease diagnosis [9,10,11,12], explosives detection [13,14,15] and spaceflight applications [16]. This paper is mainly about E-nose research in indoor pollutant gas detection.

With the modern improvement of life quality, people demand higher indoor air quality. Because most of the time during a person’s life is spent indoors, it is necessary for people’s health to be able to detect indoor pollutant gases, which mainly dfff4 include benzene (C_6_H_6_), toluene (C_7_H_8_), and so on. They are hard to detect, and will do the body harm after long-term intake, so a high recognition rate of gas detection equipment is vital. Consequently, there has been a resurgence of interest in developing measuring techniques for air quality monitoring. Our previous work has proved that E-noses are an effective way to classify indoor pollutant gases [17,18].

When an E-nose is applied to detect an indoor pollutant gas, in addition to some factors like the quality of samples, the parameter settings of the algorithm system also plays a significant role in the performance of the E-nose. In this paper, we employ support vector machine (SVM) to distinguish indoor pollutant gases and two of its parameters need to be optimized for this purpose. To make the performance of E-nose ideal, some optimization algorithms need be employed to set the value of its parameters.

Genetic algorithm (GA) is a robust and frequently-used stochastic meta-heuristic search method for global optimization in a large search space. The genetic information is encoded as a genome. The genome is implemented in an uncommon way that permits asexual reproduction which leads to offspring that are genetically the same as the parent. Meanwhile asexual reproduction can exchange and reorder chromosomes, giving birth to offspring containing a hybridization of genetic information from all parents. This operation is frequently called crossover because of the chromosomes’ crossover when swapping genetic information. To evade premature convergence, mutation emerges to increase the diversity of the population. Particle swarm optimization (PSO) is an evolutionary computation algorithm based on swarm intelligence theory. The algorithm comes from the simulation of the bird predation behavior, and its emphases lie in the cooperation and competition between individuals. Quantum-behaved particle swarm optimization (QPSO) is a new PSO algorithm and is inspired by the consideration of quantum mechanics with the PSO algorithms. It is superior to the traditional PSO algorithm not only in search ability, but also in its accuracy. Particles of this model based on a delta potential well can appear in any point of search space with a certain probability.

According to the research on optimization algorithms during the past twenty years, there are some optimization methods which have been introduced to E-nose research. They mainly include GA [19,20,21], PSO [22,23] and QPSO [24,25]. A new integer-based GA approach [19] was used to enhance the performance of E-noses by sensor selection. The PSO [22] was posed to analyze signals of wound infection detection based on an E-nose. A new feature selection method based on QPSO was proposed to optimize the gas sensor array and reduce the dimensions of the feature matrix [24]. Furthermore, an enhanced QPSO based on genetic algorithm (G-QPSO) [25] was employed to improve the performance of the sensor array and the E-nose classifier. However, up to now, we haven’t seen anyone apply the krill algorithm (KH) to E-noses. Considering the good performance of KH in global optimization, we propose to apply it to the E-nose in indoor pollutant gas classification.

The KH [26] algorithm was first proposed by Gandomi and Alavi in 2012. It has excellent local exploitation ability, while its global exploring ability is not strong, so it easily falls into local minima and has a slow convergence speed. To solve this issue, Wang et al. presented a new krill migration (KM) operator [27] which updated the krill to deal with optimization problems more efficiently. In [28] harmony search (HS) is applied to mutate between krill during the process of krill updating instead of using physical diffusion to improve the convergence speed of the algorithm and the ability to jump out of local extreme. In 2014, an improved KH [29] was proposed with linear decreasing Ct which can adjust the balance between exploration and exploitation. To overcome the poor exploitation of the KH algorithm, Wang et al. presented a hybrid differential evolution KH (DEKH) [30] method for function optimization. The IKH [31] method using a new Lévy flight distribution and elitism scheme to update the KH motion calculation was proposed by Guo et al.

In this paper, a new algorithm approach for air quality detection with an E-nose is presented. For an E-nose, the algorithm parameter setting plays an important role in practical application performance. So far, most of the E-noses are optimized by PSO, QPSO and GA. In order to make a further contribution to E-nose research and explore different optimization algorithms in the application of E-noses, we decide to adopt the KH as the optimization method of an E-nose and apply it to detect indoor harmful gaseous pollution. For the better performance of the E-nose, based on KH with an updated crossover operator [32] (defined as the standard KH in the paper), we propose a novel way of computing the decision weighting factor for KH to guarantee the krill are diversiform at the early stage of iterations, and have good local searching ability performance at the later stage of iteration. The added decision weighting factor updates the krill’s position according to the influence of other individuals and their foraging behavior under different iterations. The proposed EKH method is verified via the data obtained by our self-made E-nose.

The rest of the paper is structured as follows: materials and experiments are described clearly in Section 2. An overview of the standard KH algorithm and the proposed EKH are discussed in Section 3. Our method is compared with other optimization techniques (including CKH, KH, QPSO, PSO and GA) and the classification results presented, analyzed and compared in Section 4. Finally, the conclusions of this paper are drawn in Section 5.

## 2. Materials and Experiments

The data used in the paper were obtained by a self-made E-nose, whose detailed information can be found in our previous publication [9]. However, to make the paper self-contained, the system structure and experimental setup are briefly repeated in the following subsections.

### 2.1. Target Gas and Experimental Setup

Four common kinds of indoor pollutant gases including C_6_H_6_, C_7_H_8_, CH_2_O and CO are the target gases which will be distinguished by the E-nose in our project. The sensor array of the E-nose presented in this paper contains five sensors: three metal oxide semi-conductor gas sensors (TGS 2201, TGS 2620 and TGS 2602 purchased from Figaro Company (Osaka, Japan); the TGS 2201 has two outputs defined as TGS 2201A and TGS 2201B), one temperature sensor and one humidity sensor. The sensitive characteristics of the three gas sensors are shown in Table 1.

**Figure 1:**
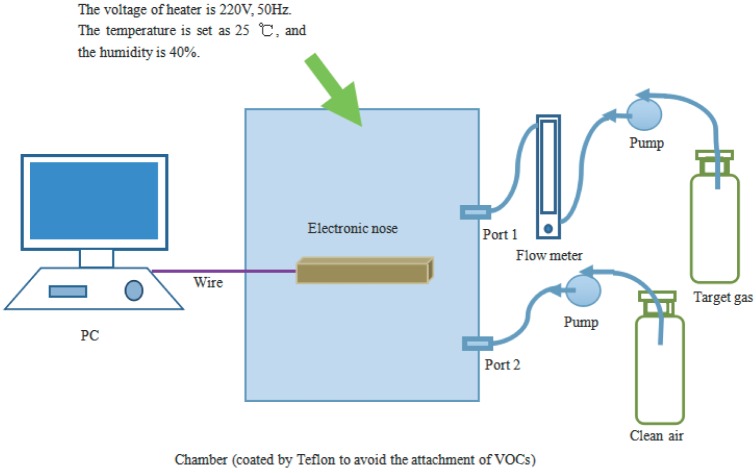
Schematic diagram of the experimental system. The experimental platform mainly consists of the E-nose system, a PC, a temperature-humidity controlling chamber, a flow meter and an air pump. There are two ports on the sidewall of the chamber, and the target gas and the clean air are put into the chamber through ports 1 and 2, respectively.

**Figure 2:**
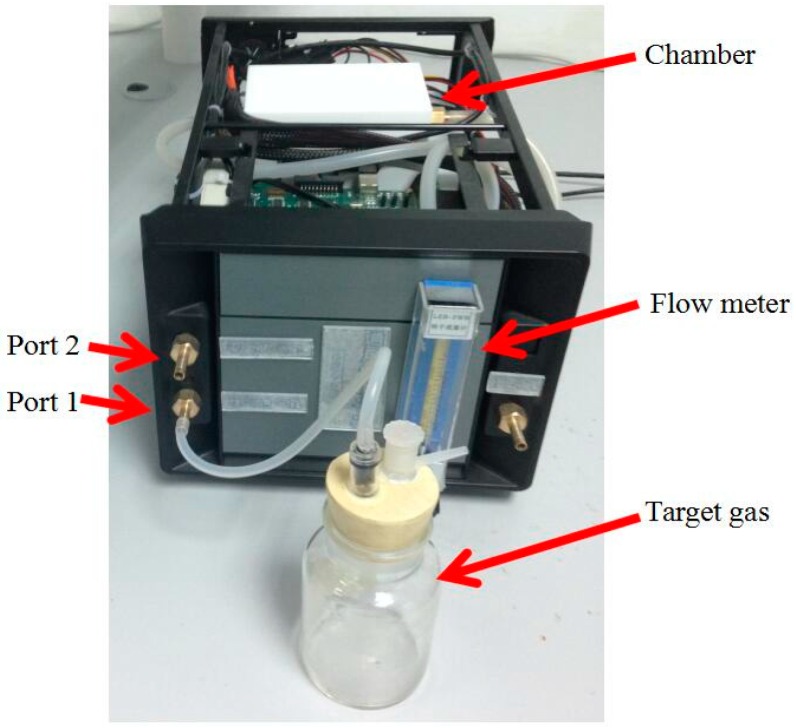
Image of the experimental setup. Data collected from the sensor array can be saved on a PC through a joint test action group (JTAG) port with its related software.

A 12-bit analog-digital converter (A/D) is used as interface between the sensor array and a field programmable gate array (FPGA) processor. The A/D converts analog signals taken from the sensor array into digital signals, and the sampling frequency is set as 1 Hz. As shown in Figure 1, the experimental platform mainly consists of the E-nose system, a PC, a temperature-humidity controlling chamber (coated with Teflon to avoid the attachment of VOCs), a flow meter and an air pump. There are two ports on the sidewall of the chamber, and the target gas and the clean air are put into the chamber through ports 1 and 2, respectively. Data collected from the sensor array can be saved in a PC through a joint test action group (JTAG) port with its related software. An image of the experimental setup is shown in Figure 2.

### 2.2. Sampling Experiments and Data Pre-Processing

Before sampling experiments, we firstly set the temperature and humidity of the chamber as 25 °C and 40%, respectively. Then we can begin the gas sampling experiments. A single sampling experiment will implement the following three phases:

Phase 1: All sensors are exposed to clean air for 2 min to obtain the baseline;
Phase 2: Target gas is introduced into the chamber for 4 min;
Phase 3: The array of sensors is exposed to clean air for 9 min again to wash the sensors and make them recover their baseline.

**Figure 3:**
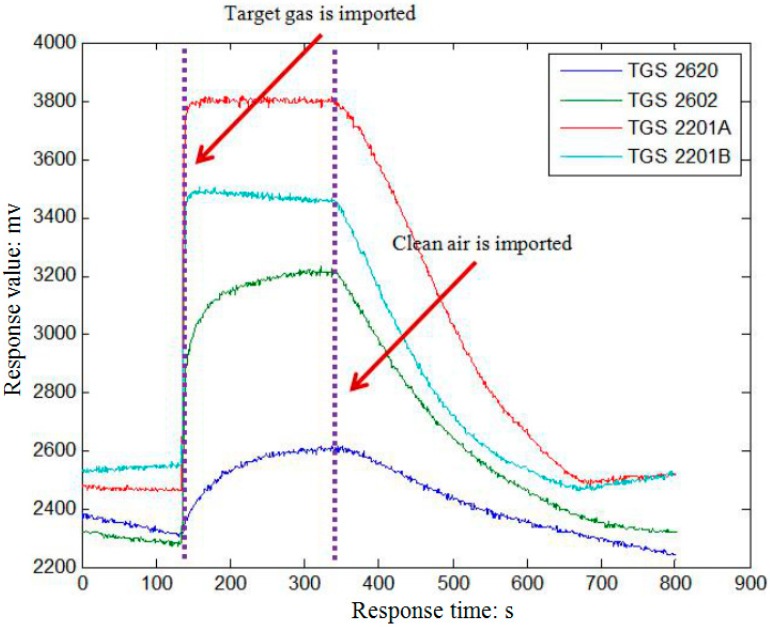
Response of the sensors array. It illustrates the response of sensors when formaldehyde is introduced into the chamber.

Figure 3 illustrates the response of sensors when formaldehyde is introduced into the chamber. We can see that each response curve rises obviously from the third minute when the target gas begins to pass over the sensor array, and recovers to the baseline after the seventh minute when clean air is conveyed to wash the sensors.

To get the real concentration of gas in the chamber, we extract each gas from the chamber and take it into a gas bag. Then a spectrophotometric method is employed to determine the concentration of formaldehyde and carbon monoxide, and the concentration of benzene and toluene are determined by gas chromatography (GC). For each gas, there are 12, 11, 21 and 29 concentration points, respectively, and 12 sampling experiments are made on each concentration point. The real concentration and the numbers of samples of the four kinds of gases are shown in Table 2. We set different concentrations of gas mainly in order to improve the generalization of algorithm, and try to avoid the misjudgment of the results when the concentration of the test gas is not the same concentrations with the gas of experiment. And the purpose of our work is to distinguish four indoor pollutant gases with E-nose.

Then the maximum value of the steady-state response of sensors is extracted to create the feature matrix of the E-nose. There are 1932 samples in this matrix and the dimension of each sample is 4. We randomly select 70% of the samples of each gas to establish the training data set, and the rest are used as the test data set. Detailed information is shown in Table 3.

## 3. KH Algorithm

### 3.1. Overview of Standard KH Algorithm

KH is a new generic stochastic optimization approach for the global optimization problem. It is inspired by the behavior of krill swarms. When hunting for the food and communicating with each other, the KH approach repeats the implementation of the three movements and follows search directions that enhance the objective function value. The time-relied position is mostly determined by three movements:

- Foraging action;
- Movement influenced by other krill;
- Physical diffusion.

Regular KH approach adopts the Lagrangian model as shown in the following expression:

(1) $$\frac{dx_{i}}{d_{t}}=N_{i}+F_{i}+D_{i},$$

where *N_i_*, *F_i_* and *D_i_* denote the foraging motion, which is influenced by other krill and the physical diffusion of krill *i*, respectively. The first motion *F_i_* covers two parts: the current food location and the information about the previous location. For krill *i*, we formulate this motion as below:

(2) $$F_{i}=V_{f}\beta_{i}+w_{f}F_{i}^{old}$$

where:

(3) $$\beta_{i}=\beta_{i}^{food}+\beta_{i}^{best}$$

and *V_f_* is the foraging speed, *w_f_* is the inertia weight of the foraging motion in (0, 1), is the last foraging motion.

The direction led by the second movement *N_i_* , *a_i_* is estimated by the three effects: target effect, local effect, and repulsive effect. For a krill *i*, it can be formulated as below:

(4) $$N_{i}^{new}=N^{max}a_{i}+w_{n}N_{i}^{old}N_{i}^{new}=N^{max}a_{i}+w_{n}N_{i}^{old},$$

where *N^max^* is the maximum induced speed, *w_n_* is the inertia weight of the second motion in (0, 1), is the last motion influenced by other krill.

For the *i-*th krill, in practice, the physical diffusion is a random process. This motion includes two components: a maximum diffusion speed and an oriented vector. The expression of physical diffusion can be given below:

(5) $$D_{i}=D^{max}\delta ,$$

where *D^max^* is the maximum diffusion speed and δ is the oriented vector whose value is a random number between −1 and 1.

According to the three above-analyzed actions, the time-relied position from time *t* to t+Δt can be formulated by the following equation:

(6) $$X_{i}\left(t+\Delta t\right)=X_{i}\left(t\right)+\Delta t\frac{dx_{i}}{dt}.$$

For more detailed information about the KH method readers may referred to [26].

### 3.2. EKH

**Figure 4:**
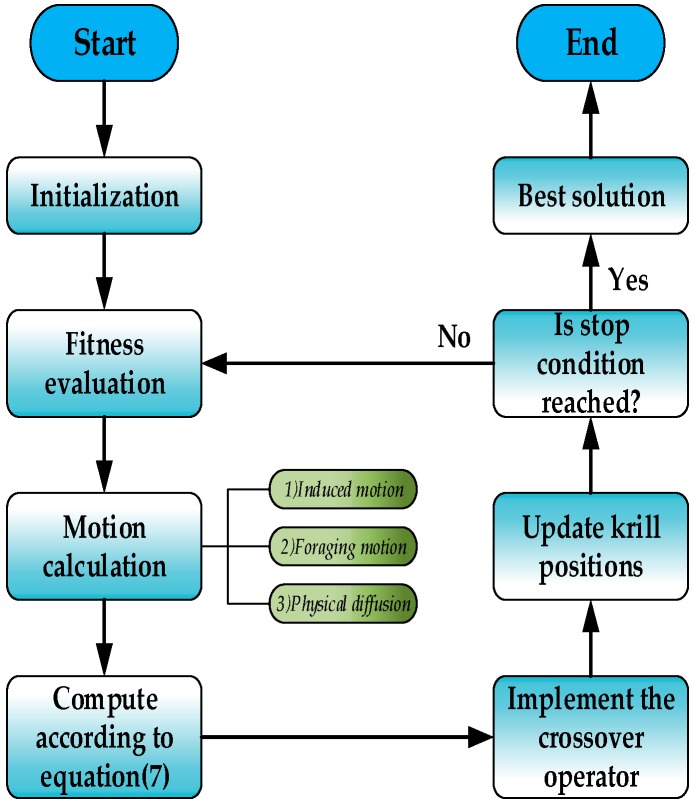
Simplified flowchart of EKH. In addition to the basic steps of krill algorithm, the flowchart of EKH also includes the novel computing way of decision weighting factor used in Equation (7) and an updated crossover operator.

It has been proved that KH is an effective method in exploitation. However because the search relies fully on randomness, it cannot converge rapidly. In the group strategy optimization algorithm, the number of iterations could affect the performance of the algorithm, and sometimes even determines whether we can find the global optimal point. We should also also consider the factor of time (the optimization process should be as quick as possible), so we present a novel way of computing the decision weighting factor to give KH better global searching ability performance and a higher convergence speed. Its equation is shown as Equation (7):

(7) $$\frac{dx_{i}}{dt}=\frac{MI-I}{MI}F_{i}+\frac{I}{MI}N_{i}+D_{i},$$

where *MI* is the maximum iteration, and *I* is the current number of iterations. At the early stage of iterations, (*MI – I*)/*MI* > *I/MI*, their foraging actions should have more influence on their decisions for the next position. Because each krill doesn’t know the correct direction, that krill start with their own feelings can effectively help them avoid premature. At the later stage of iterations, (*MI – I*)/*MI* < *I/MI*, the experience of other krill has more influence when they update their next position. After all, the correctness of the group direction tends to be higher than that of the individuals. Finally, we define the KH with an updated crossover operator as the standard krill swarm algorithm. The method we proposed as the enhanced KH (EKH). The basic framework of the EKH method and its responding flowchart are shown in Algorithm 1 and Figure 4.
**Algorithm 1.** EKH algorithm***Begin*** ***Step 1: Initialization.** Initialize the Iteration counter I=1, the population P of NP krill, V_f_, D^max^ and N^max^.* ***Step 2: Fitness calculation.** Calculate fitness for each krill according to its initial position.* ***Step 3: While** I < Maximum Iteration **do*** *Sort all the population according to their fitness.****for***
*i=1:NP (all krill) **do*** *Perform the following motion calculation.* *Motion induced by other individuals* *Foraging motion* *Physical diffusion* *Compute dx_i_/dt according to Equation (7).* *Implement the crossover operator.* *Updating the krill individual position in the search space.* *Calculate fitness for each krill according to its new position.****end for***
*i**I = I+1.* ***Step 4: end while******End.***

## 4. Results and Discussion

To evaluate the effectiveness of the optimization algorithm we analyze the discrimination of four different gases with our self-made E-nose. We compare EKH with QPSO, PSO and GA which have been frequently used in E-noses. We also compare EKH with the standard KH and the chaotic KH (CKH) [33]. In CKH, various one-dimensional chaotic maps are employed in place of the parameters used in the KH to accelerate the convergence speed of it. According to the results of [33], we choose Singer map as the proper chaotic map to form the best CKH. It is shown in Equation (8).

Singer map:

(8) $$x_{k+1}=u(7.86x_{k}-23.31x_{k}^{2}+28.75x_{k}^{3}-13.30x_{k}^{4}),$$

The parameter setting in all experiments for each algorithm is shown in Table 4.

**Figure 5:**
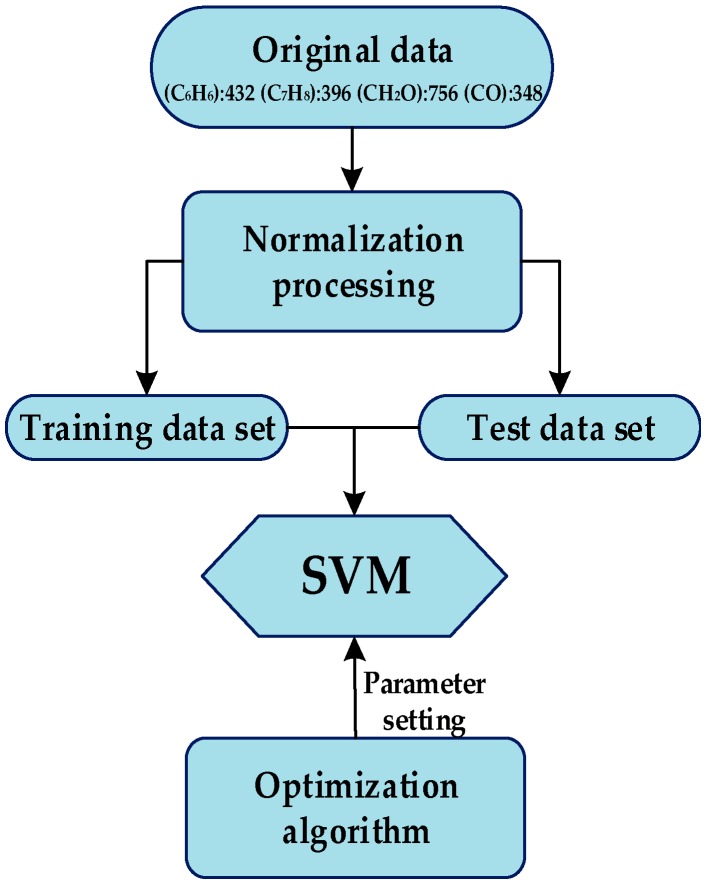
Flow diagram of experiment. Firstly, a normalization processing is made. Then the support vector machine (SVM) is employed as the classifier. Its two parameters are optimized by the six considered optimization algorithms. All of the optimization algorithms to optimize parameters of SVM are mainly based on the training data set.

The flow of data processing is as follows: firstly, a normalization processing is performed. Then the SVM [34,35] is employed as the classifier. Its two parameters are optimized by the six considered optimization algorithms. The flow diagram of the experiment is shown in Figure 5. All of the optimization algorithms to optimize parameters of SVM are mainly based on the training data set. Finally SVM will distinguish the class label of each sample in test data set with the knowledge it has learned, and the ratio (the number of points distinguished directly to the number of all points in test data) will be used to evaluate the performance of the different optimization algorithms.

There are two parameters need to be set in SVM (the spread factor of the Gaussian RBF kernel function and the penalty factor), so krill group search in the two-dimensional space. Each kind of particle number optimization algorithm is set to 30, and in order to compare the differences between the algorithms, we set the number of iterations, to 50, 200 and 400, respectively. To make sure the accuracy of experimental results is correct, each program was repeated 10 times. Then we take the ten times’ classification accuracy (the training data set and test data set) in maximum, minimum and average value as a reference to evaluate the performance of the six kinds of optimization algorithms. Table 5, Table 6 and Table 7 show the classification accuracy of the different optimization algorithms with the number of iterations set as 50, 200 and 400. The best classification accuracy of the four kinds of gases and all the classification accuracies with different optimization algorithms are shown in Table 8, Table 9, Table 10, Table 11 and Table 12. To make our research more persuasive, we use the 10-fold cross validation method to train and test the data and the particle number is set to 50. All the results of algorithms after using 10-fold cross validation are shown in Table 13, Table 14 and Table 15. It also reachs the same conclusion that the EKH has the best performance. When the particle number is set to 50, the results with the number of iterations set as 50, 200 and 400 are shown in Table 16, Table 17 and Table 18. In Table 18, the standard deviations (SD) of each kind of algorithm after running for 100 times are shown to evaluate the performance of the algorithm more precisely.

EKH and CKH are both the enhanced optimization algorithms based on the KH. and comparing the results of EKH, CKH and KH from Table 5, Table 6 and Table 7, we can find that the best results are obtained by EKH. In the case of a higher number of iterations, the CKH performs a little better than KH, however, when it comes to the maximum, minimum or the average value of classification accuracy, the EKH significantly outperforms CKH and KH. This verifies that the EKH we proposed is more appropriate than CKH in the application of E-noses in gas identification. What’s more, it’s easy to see whatever the number of iterations given, the worst classification accuracy of EKH is higher than the best classification accuracy of CKH and KH. The results when the number of iterations is 200 and 400 are very close. All of these results prove that the global searching and convergence of EKH has been improved with the influence of the novel way of computing the decision weighting factor.

Comparing the EKH with different algorithms (QPSO, PSO and GA), it can be found from Table 5, Table 6 and Table 7 that GA has the worst performance, while PSO and QPSO are better. In terms of the truth that the EKH has the highest classification accuracy in the same iterations, relative to the three other algorithms, once again it proves that the krill algorithm can be applied well in E-noses.

**Figure 6:**
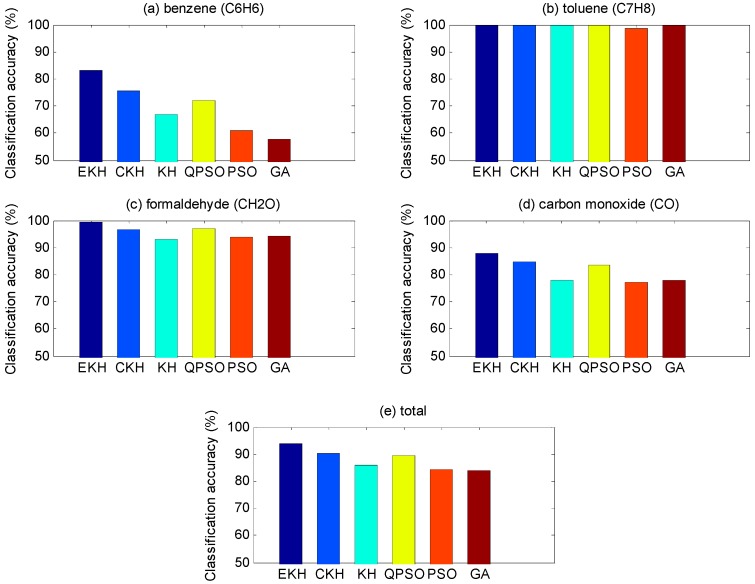
Classification accuracy based on training set. The classification results of training set based on different optimization algorithms and the discrepancy between each other. The iteration is 400 and the particle number is 30.

**Figure 7:**
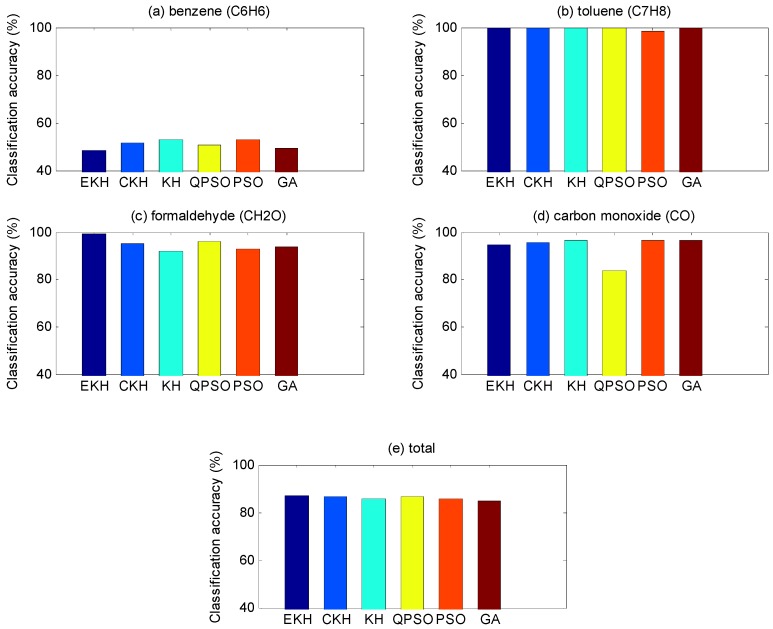
Classification accuracy based on test set. The classification results of test set based on different optimization algorithms and the discrepancy between each other. The iteration is 400 and the particle number is 30.

Table 8, Table 9, Table 10 and Table 11, respectively, show the classification accuracy of four kinds of gases being measured under the condition that total classification accuracy is best. We can also draw a conclusion from the data that C_6_H_6_ is harder to distinguish compared with other gases. For EKH, except for the fact the recognition rate of C_6_H_6_ of the test set is a bit low, the other results are very reasonable, not only in terms of itself but also with other algorithms. According to the results in Table 18, we can know that the SD of EKH is the smallest. It suggests that the EKH result is more stable. That is to say, the EKH is better in average performance than KH and other algorithms. In Figure 6 and Figure 7, through the colorful bar chart, we can clearly see the classification results of training set and test set based with different optimization algorithms and the discrepancies between each other.

## 5. Conclusions

There is no doubt that E-noses play an important role in the field of environmental monitoring and control of pollution emissions. In this experiment an E-nose is applied to distinguish four kinds of indoor pollutant gases. We all know that an E-nose device which has a high recognition rate for pollutant gases is significant to the improvement of the quality of people’s indoor life, so we have undertaken further research on the E-nose algorithm to improve its gas recognition rate.

An E-nose mainly consists of an array of sensors and an appropriate pattern-recognition system. The pattern-recognition system has a significant effect in helping E-noses make a correct decision via the algorithm. Furthermore, the value of parameters determines the performance of the pattern recognition system, so some algorithms must be employed to select the appropriate parameters. KH is a new optimization algorithm put forward in recent years, that has not been applied yet to the E-nose technology, so we have creatively applied the krill algorithm to the classification problem of E-noses for indoor pollutant gases. Considering the practical application we propose an EKH based on a novel way of computing the decision weighting factor.

The KH technique has a good performance in exploitation, but it cannot always converge rapidly to find the global optimum. In this paper, we present an effective EKH algorithm based on a novel way of computing decision weighting factors and apply it to optimize the parameters of our self-made E-nose which is employed to distinguish different indoor pollutant gases. Through comparing EKH with other optimization methods, we find that the performance of EKH is better than KH, CKH, QPSO, PSO and GA. We can draw the conclusion according to the results that EKH is an ideal optimization method for E-noses in distinguishing indoor pollutant gases. Of course, we will continue to further study the krill algorithm in the future, and we believe the performance of E-nose will be further improved.

## Figures and Tables

**Table 1:** Main sensitive characteristics of gas sensors.

Sensors	Main Sensitive Characteristics
TGS2201	Carbon monoxide, nitrogen dioxide, nitric oxide,
TGS2620	Carbon monoxide, VOCs, methane, ethanol, isobutane,
TGS2602	Ammonia, VOCs, toluene, ethanol, hepatic gas, formaldehyde

Note: the responses of these three sensors is non-specific. Besides their main sensitive gas listed in Table 1, they are also sensitive to other gases.

**Table 2:** Concentration of the target gas.

Gas	Concentration Range (ppm)	Number of Samples
Benzene	[0.1721, 0.7056]	432 (12 × 12)
Toluene	[0.0668, 0.1425]	396 (12 × 11)
Formaldehyde	[0.0565, 1.2856]	756 (12 × 21)
Carbon monoxide	[4, 12]	348 (12 × 29)

**Table 3:** Amount of samples in training set and test set.

Gas	Training Set	Test Set
Benzene	288	144
Toluene	264	132
Formaldehyde	504	252
CO	232	116
All-4	1288	644

**Table 4:** Parameter setting.

Algorithms	Parameters
EKH	The foraging speed *V_f_* = 0.02, the maximum diffusion speed *D^max^* = 0.005, the maximum induced speed *N^max^* = 0.01
CKH	The foraging speed *V_f_* = 0.02, the maximum diffusion speed *D^max^* = 0.005, the maximum induced speed *N^max^* = 0.01
KH	The foraging speed *V_f_* = 0.02, the maximum diffusion speed *D^max^* = 0.005, the maximum induced speed *N^max^* = 0.01
QPSO	An inertial constant = 0.3, a cognitive constant = 1, and a social constant for swarm interaction = 1
PSO	An inertial constant = 0.3, a cognitive constant = 1, and a social constant for swarm interaction = 1
GA	Roulette wheel selection, single point, two point and uniform crossover with a crossover probability of 0.6, and a mutation probability of 0.01

**Table 5:** Classification accuracy of different optimization algorithm (%).

		EKH	CKH	KH	QPSO	PSO	GA
Training set	best	91.85	85.33	83.85	81.21	81.37	83.79
	mean	90.94	79.71	82.17	80.20	79.01	82.92
	worst	90.37	76.78	80.67	78.73	77.64	82.14
Test set	best	87.89	85.40	85.55	83.39	83.85	80.90
	mean	86.80	82.81	84.52	83.13	82.66	80.25
	worst	86.18	81.21	83.38	82.30	81.99	79.43

Note: the iterations are 50.

**Table 6:** Classification accuracy of different optimization algorithm (%).

		EKH	CKH	KH	QPSO	PSO	GA
Training set	best	93.01	90.06	85.95	87.89	80.98	84.16
	mean	92.26	84.19	83.20	86.16	80.67	83.38
	worst	91.77	77.25	81.13	85.02	80.43	82.92
Test set	best	88.04	85.71	85.09	86.18	84.16	81.06
	mean	87.63	84.42	84.68	85.14	83.75	80.83
	worst	87.27	81.83	84.16	84.16	83.07	80.43

Note: the iterations are 200.

**Table 7:** Classification accuracy of different optimization algorithm (%).

		EKH	CKH	KH	QPSO	PSO	GA
Training set	best	93.79	90.45	85.95	89.59	84.39	84.94
	mean	93.01	86.85	83.39	86.39	82.61	84.89
	worst	92.08	84.47	80.05	84.32	81.06	84.78
Test set	best	88.20	86.49	85.56	86.65	85.56	84.08
	mean	87.73	85.35	84.63	85.87	84.94	83.88
	worst	87.27	84.47	83.39	85.25	84.47	83.62

Note: the iterations are 400.

**Table 8:** Classification accuracy of benzene by different optimization algorithm (%).

	EKH	CKH	KH	QPSO	PSO	GA
Training set	83.33	75.69	66.67	71.88	60.76	57.64
Test set	48.61	51.39	52.83	50.69	52.83	49.31

**Table 9:** Classification accuracy of toluene by different optimization algorithm (%).

	EKH	CKH	KH	QPSO	PSO	GA
Training set	100.00	100.00	100.00	100.00	98.48	100.00
Test set	100.00	100.00	100.00	100.00	98.48	100.00

**Table 10:** Classification accuracy of formaldehyde by different optimization algorithm (%).

	EKH	CKH	KH	QPSO	PSO	GA
Training set	99.21	96.43	93.25	97.02	93.85	94.44
Test set	99.21	95.24	92.06	96.03	92.86	93.65

**Table 11:** Classification accuracy of carbon monoxide by different optimization algorithm (%).

	EKH	CKH	KH	QPSO	PSO	GA
Training set	87.93	84.91	78.02	83.62	77.16	78.02
Test set	94.83	95.69	96.55	83.62	96.55	96.55

**Table 12:** Classification accuracy of total by different optimization algorithm (%).

	EKH	CKH	KH	QPSO	PSO	GA
Training set	93.79	90.45	85.95	89.59	84.39	84.94
Test set	87.27	86.49	85.56	86.65	85.56	84.08

**Table 13:** Classification accuracy by different optimization algorithm (%).

	EKH	CKH	KH	QPSO	PSO	GA
Training set	92.39	88.90	86.57	89.21	85.25	85.32
Test set	88.20	86.02	84.94	86.49	83.54	83.70

Note: the iterations are 50.

**Table 14:** Classification accuracy by different optimization algorithm (%).

	EKH	CKH	KH	QPSO	PSO	GA
Training set	93.56	91.38	89.13	90.45	86.96	87.34
Test set	88.51	87.42	86.18	86.96	84.63	86.02

Note: the iterations are 200.

**Table 15:** Classification accuracy by different optimization algorithm (%).

	EKH	CKH	KH	QPSO	PSO	GA
Training set	94.07	91.42	89.93	90.56	87.05	87.56
Test set	89.64	87.56	86.53	87.04	85.49	86.53

Note: the iterations are 400.

**Table 16:** Classification accuracy by different optimization algorithm (%).

	EKH	CKH	KH	QPSO	PSO	GA
Training set	92.16	88.66	86.18	88.82	84.47	84.94
Test set	87.89	85.56	84.78	85.71	82.38	81.52

Note: the iterations are 50.

**Table 17:** Classification accuracy by different optimization algorithm (%).

	EKH	CKH	KH	QPSO	PSO	GA
Training set	93.32	91.61	88.90	90.61	84.94	85.79
Test set	87.73	86.96	86.02	86.49	83.62	85.25

Note: the iterations are 200.

**Table 18:** Classification accuracy of different optimization algorithm (%).

		EKH	CKH	KH	QPSO	PSO	GA
Training set	best	94.02	91.85	89.21	91.15	85.87	85.95
	mean	93.98	91.81	89.16	91.11	85.82	85.88
	SD	0.058	0.067	0.086	0.083	0.101	0.096
Test set	best	87.58	87.42	85.71	86.80	85.17	85.40
	mean	87.53	87.39	85.67	86.74	84.95	85.25
	SD	0.075	0.084	0.098	0.093	0.326	0.183

Note: the iterations are 400.
